# Interpreting Microbial Species–Area Relationships: Effects of Sequence Data Processing Algorithms and Fitting Models

**DOI:** 10.3390/microorganisms13030635

**Published:** 2025-03-11

**Authors:** Fu-Liang Qi, Wei Deng, Yi-Ting Cheng, Xiao-Yan Yang, Na Li, Wen Xiao

**Affiliations:** 1 Institute of Eastern-Himalaya Biodiversity Research, Dali University, Dali 671003, China; qfuliang@163.com (F.-L.Q.); dengw@mail.nankai.edu.cn (W.D.); chengyt1219@dingtalk.com (Y.-T.C.); yangxy@eastern-himalaya.cn (X.-Y.Y.); 2 Collaborative Innovation Center for Biodiversity and Conservation in the Three Parallel Rivers Region of China, Dali 671003, China; 3 The Provincial Innovation Team of Biodiversity Conservation and Utility of the Three Parallel Rivers Region, Dali University, Dali 671003, China

**Keywords:** species–area relationship, microbial diversity patterns, high-throughput sequencing, model fitting

## Abstract

In the study of Species–Area Relationships (SARs) in microorganisms, outcome discrepancies primarily stem from divergent high-throughput sequencing data processing algorithms and their combinations with different fitting models. This paper investigates the impacts and underlying causes of using diverse sequence data processing algorithms in microbial SAR studies, as well as compatibility issues that arise between different algorithms and fitting models. The findings indicate that the balancing strategies employed by different algorithms can result in variations in the calculations of alpha and beta diversity, thereby influencing the SARs of microorganisms. Crucially, incompatibilities exist between algorithms and models, with no consistently optimal combination identified. Based on these insights, we recommend prioritizing the use of the DADA2 algorithm in conjunction with a power model, which demonstrates greater compatibility. This study serves as a comprehensive comparison and reference for fundamental methods in microbial SAR research. Future microbial SAR studies should carefully select the most appropriate algorithms and models based on specific research objectives and data structures.

## 1. Introduction

The Species–Area Relationship (SAR) constitutes a foundational biogeographic pattern characterizing the monotonic positive relationship between species richness and spatial scale. As one of the core principles in ecology, SARs are essential for biodiversity conservation and ecosystem management [1,2,3].

However, past research on SARs has primarily focused on animals and plants, with less attention given to microorganisms [4]. The current understanding of microbial SARs is still in its infancy, and researchers investigating microbial SARs have encountered a series of inconsistent or even contradictory results [5]. Microorganisms were once considered to lack SARs or to exhibit negligible SARs [4,6,7]. However, recent studies have shown that under specific conditions, microorganisms can also demonstrate significant SARs [8]). This situation underscores the challenges and complexities inherent in microbial SAR research. Current hypotheses suggest these discrepancies may arise from differences in study subjects (e.g., bacteria or fungi). For instance, Li and other scholars believe that although the SARs of bacteria and fungi are similar, their underlying mechanisms differ [9]. Other research attributes these differences to environmental factors; for example, Moradi et al. suggest that the SAR slope is positively influenced by temperature and soil nitrogen, which decreases with increasing altitude [10]. Additionally, some studies indicate that the choice of fitting model may lead to variations in results. For example, Zhang et al. found that the power-law model cannot accurately evaluate SARs and lacks sufficient validation [11]. We posit that in microbial SAR studies, processing algorithms for high-throughput sequencing data could significantly influence results, and beyond model limitations, algorithm compatibility issues may further contribute to variability.

With the advent of high-throughput sequencing, significant progress has been achieved in rapidly monitoring microbial diversity, leading to substantial advancements in microbial SARs. To convert the vast amounts of unknown sequence data generated from sequencing into reliable species richness estimates, complex algorithmic processing is required. Currently, widely adopted algorithms include UPARSE [12], DADA2 [13], UNOISE3 [14], and Deblur [15]. These algorithms exhibit considerable divergence in their approaches to quality control, denoising, and clustering. For instance, UNOISE3 and Deblur typically eliminate low-abundance taxa, resulting in more conservative outcomes, whereas UPARSE and DADA2 are more likely to retain these taxa, which can lead to increased noise or false positives [16,17,18]. Such methodological variations directly impact the interpretation of microbial SARs. Crucially, a lack of standardized algorithms in current research may not only complicate the interpretation of microbial patterns but also affect the accuracy of SAR model fitting, ultimately compromising the reliability of research findings.

The methodological outcomes of sequence processing algorithms exert profound influences on SAR research, and the differences between algorithms may lead to incompatibility with various fitting models. When conducting SAR model fitting, the effectiveness of the model is closely related to the data structure. At the current stage of SAR research, numerous fitting models have been developed, including power relationships, exponential decay, logarithmic relationships, and proportional relationships, all aimed at accurately describing the complex interplay between biodiversity and spatial scale [3,19]. However, the selection of these models often depends on the researcher’s interpretation of the data and the specific research questions, with each model emphasizing different aspects. This can lead to varying impacts of different models on the same dataset [20]. Different processing algorithms can lead to significant variations in the richness and diversity estimates of microbial data, resulting in diverse microbial diversity data structures. The data structures required by various SAR fitting models are often inconsistent, which directly impacts the ecological characteristics that need to be captured during the fitting process. Therefore, algorithms must accurately reflect their data characteristics, and the selection of SAR models should, in turn, guide the choice of specific algorithms to meet application needs. This approach ensures a more accurate representation of microbial ecological characteristics. If compatibility between algorithms and models is not taken into account, the interaction between the two may obscure our understanding of the shape of Species–Area Relationships, leading to confusion in microbial SAR research.

To investigate the impact of different processing algorithms on the SARs of microorganisms, as well as the compatibility between these algorithms and various models, this study analyzed eight microbial communities with well-defined characteristics. Initially, the raw data were processed using four algorithms: UPARSE, UNOISE3, DADA2, and Deblur. Following this, a power model was employed after logarithmic transformation for fitting and mutual comparison to assess how different algorithms influenced the results. Subsequently, 20 commonly used fitting models in SAR studies were applied to the data obtained from the four algorithms to evaluate whether the choice of model would affect the fitting outcomes and to analyze the compatibility between models and algorithms. This study systematically investigates the dual impact mechanisms of high-throughput sequencing algorithms and fitting model selection on microbial SAR research, aiming to fill the methodological gap in microbial macroecology. This work not only potentially resolves the incomparability of results caused by mismatches between data processing and model selection but, more importantly, provides critical theoretical support for developing standardized analytical procedures in microbial research.

## 2. Materials and Methods

### 2.1. Data Source

This study utilized eight original datasets from Deng’s paper (DOI: 10.3389/fmicb.2023.1093695) and the GSA database (data accession number CRA008829), available at https://ngdc.cncb.ac.cn/gsa/browse/CRA008829, accessed on 23 May 2023 [8].

In this study, microbial data were collected by exposing eight 60 × 60 cm sterile filter papers to ambient conditions for 12 h. Area gradients were generated through the cumulative stacking of filter papers, enabling investigation of the Species–Area Relationship (SAR).

This dataset features microbial communities initiated under identical baseline conditions, systematically controlling for methodological artifacts (sampling protocols), environmental variability, and successional dynamics that typically confound SAR detection. The experimental design demonstrates high reproducibility [8,21,22], thereby providing robust scientific support for our research objectives while minimizing systematic bias.

### 2.2. Data Processing and Species Classification

This study processed high-throughput sequencing data from eight samples using four algorithms (UPARSE, DADA2, UNOISE3, and Deblur) for quality control, trimming, denoising, assembly, and removal of chimeras. The processed data generated a sequence variant feature table. Representative sequences were then aligned with the SILVA_16S_v123 database to obtain species classification information for the samples [23].

### 2.3. The Combination of Species–Area Relationship Data

Following Deng et al. [8], eight filter paper samples were randomly selected using the sample function in R. These samples corresponded to filter paper areas of 3600, 7200, 10,800, 14,400, 18,000, 21,600, 25,200, and 28,800 cm^2^. The procedure was repeated eight times to generate eight distinct island models.

### 2.4. Linear Transformation and Fitting of the Power Model

The logarithmic form (log-log power) of the power model was used to fit the SAR. The model is expressed as follows:

log(Richness) = z (slope) ∗ log(area) + c

Here, *z* represents the slope, and *c* represents the intercept. By standard linear regression, the relationship between species richness and area were fitted, and the model’s R^2^, slope *z*, and *p*-value were calculated and recorded to evaluate the significance of SAR. This method follows the study by Rosenzweig [24].

### 2.5. Diversity Analysis and Visualization

The vegan and ggplot2 packages in R were used to generate rarefaction curves and species richness curves for four algorithms. Euclidean distances between samples were calculated and visualized through box plots to compare the effects of these algorithms on sample richness.

### 2.6. Fitting and Selection of SAR Models

The 20 SAR models available in the R package sars were utilized to fit each dataset [25]. Models with R^2^ values exceeding 0.6 were first eliminated from the fitting results. The AICc values of the remaining models were then compared, and the model with the lowest AICc value was selected as the best-fitting candidate.

R^2^ threshold of 0.6: In ecological studies, the influence of multiple environmental factors and ecological processes on biodiversity often results in R^2^ values that are not particularly high. Nevertheless, the model may still hold significant ecological relevance. Therefore, selecting 0.6 as a more stringent threshold can ensure that the model possesses adequate explanatory power, meaning it can account for a substantial portion of the variation in the relationship between species richness and area. This approach also helps prevent the loss of valuable ecological information due to the complexities of multiple interacting factors [26].

AICc value: The AICc (Corrected Akaike Information Criterion) measures the relative information loss of a model, with a lower AICc value indicating a better fit to the data while minimizing the risk of overfitting. Compared to the AIC, the AICc imposes a more stringent penalty on models with a greater number of parameters, making it particularly suitable for scenarios with smaller sample sizes to mitigate overfitting. In this study, the sample size is limited, and utilizing AICc allows for a more accurate assessment of the model’s fit [27,28]

### 2.7. Visualization of the Best Models

Bubble plots for the top models selected from each algorithm were created using GraphPad Prism 9 software. The size of the bubbles indicates the relative performance of each model, offering an intuitive comparison of the best models across the four algorithms.

## 3. Results

### 3.1. Impact of Different Algorithms on Microbial SARs and Species Diversity Comparison

**Figure 1:**
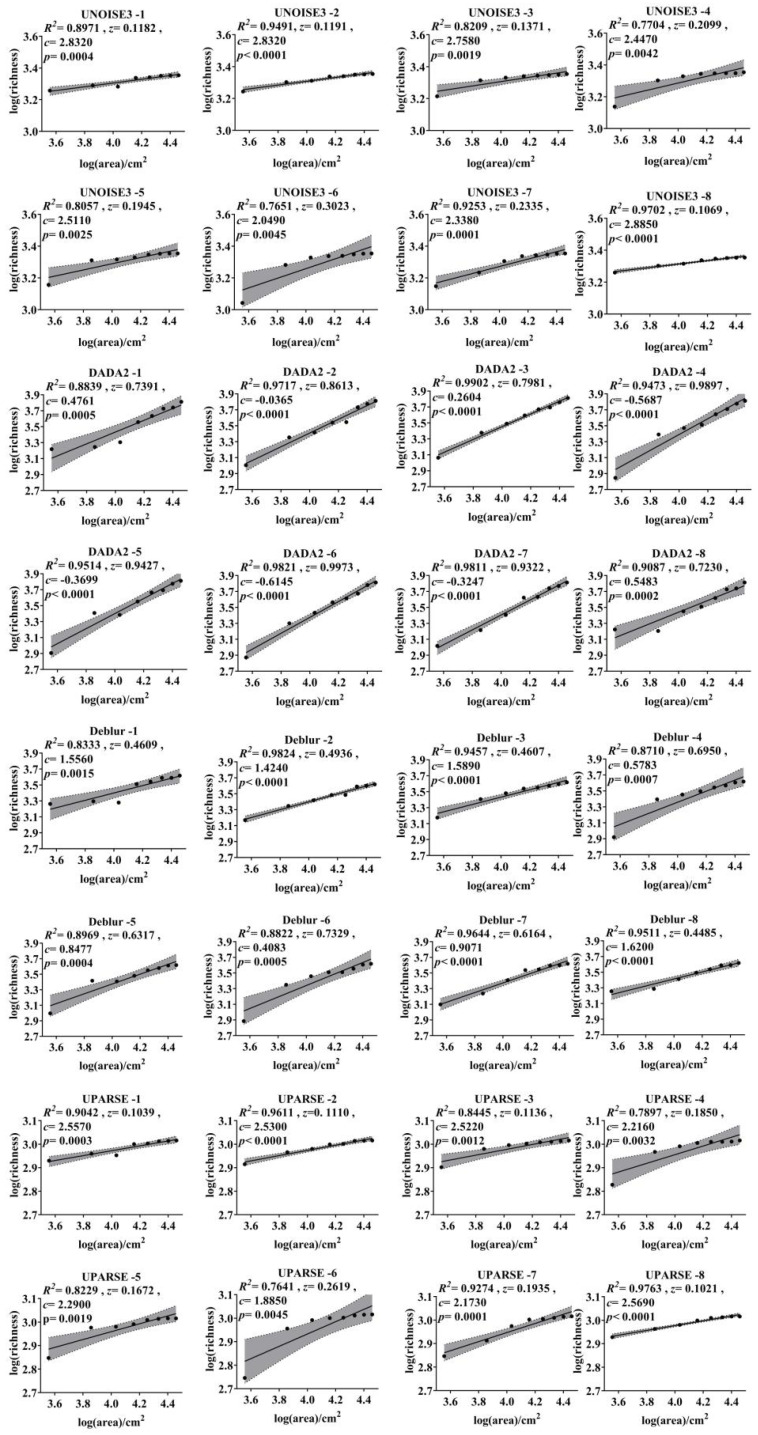
SAR curves under different algorithms.

**Figure 2:**
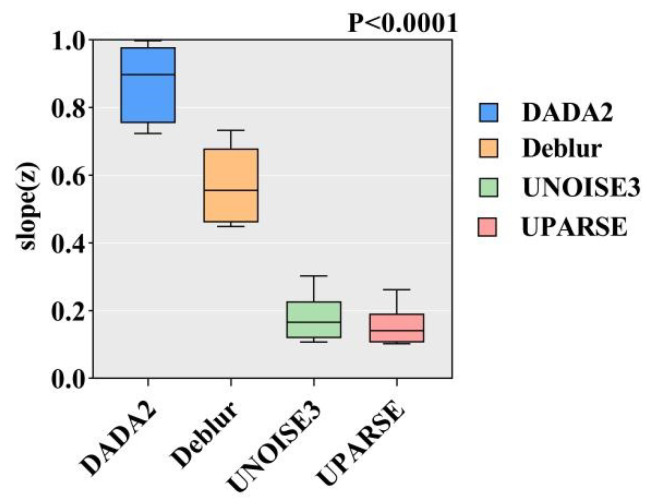
The slopes of the SAR curves under four different algorithms. The slope of the SAR curve, derived from fitting the power model, was analyzed along with *p*-values obtained through a Kruskal–Wallis test.

The power model fitted using linear transformation for the eight datasets across four algorithms indicates that the species richness of the eight filter paper groups is significantly positively correlated with the sampling area. Slope ranges for the eight samples under the four algorithms are as follows: 0.1039–0.7391, 0.1110–0.8613, 0.1136–0.7981, 0.1850–0.9897, 0.1672–0.9427, 0.2619–0.9973, 0.1935–0.9322, and 0.1021–0.7230, with an overall fluctuation range of 0.6209–0.8047 (Figure 1). The DADA2 algorithm yields the highest fitting line slopes (0.7230–0.9973), followed by the Deblur algorithm (0.4485–0.7329), whereas the UNOISE3 (0.1069–0.3023) and UPARSE (0.1021–0.2619) algorithms exhibit comparatively lower values. The sixth dataset shows the highest slope across all algorithms, while the eighth dataset displays the lowest. Kruskal–Wallis test results indicate statistically significant differences among the four algorithms (*p* < 0.0001) (Figure 2).

**Figure 3:**
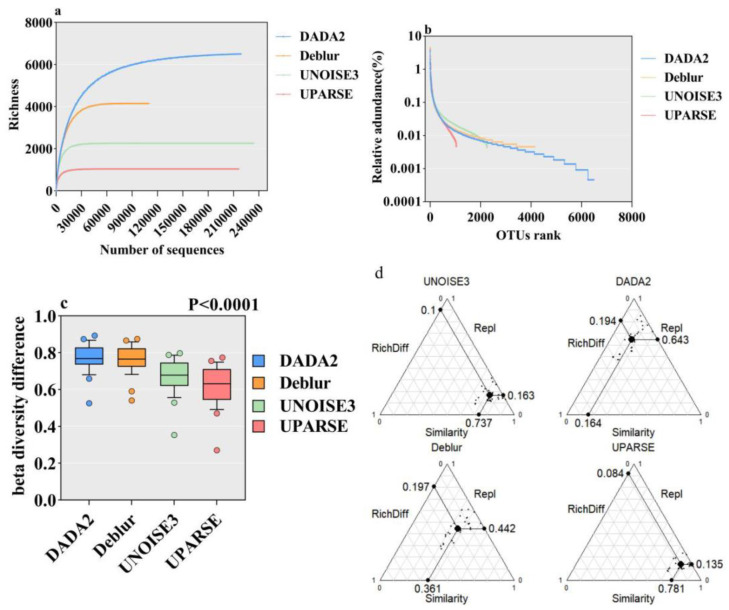
Comparison of alpha diversity and beta diversity among four algorithms. (**a**) displays the overall rarefaction curves for the four algorithms based on species richness; (**b**) illustrates the overall species abundance rank curve for the four algorithms; (**c**) presents a beta diversity box plot comparing samples across the four algorithms, with *p*-values derived from a Kruskal–Wallis test indicating differences; (**d**) conducts a beta diversity partitioning analysis on eight sample datasets from the four algorithms, where each black dot represents the comparison value between two samples. The positions of the dots are determined by the Richness Difference, Replacement, and Similarity, with the sum of each triplet equaling one. Larger black dots indicate the centroids of the points, representing the average values of Richness Difference, Replacement, and Similarity.

Comparing species diversity across four algorithms, initially, the rarefaction curves for all four algorithms tend to plateau (Figure 3a), with DADA2 demonstrating the highest species richness, followed sequentially by Deblur, UNOISE3, and UPARSE. Sequence retention rates differ substantially: Deblur retains the fewest sequences, UPARSE and DADA2 exhibit intermediate retention, while UNOISE3 maintains the highest sequence count.

The abundance rank curve analysis highlights significant disparities in rare species composition. DADA2 detects the largest proportion of rare species, trailed by Deblur and UNOISE3, with UPARSE identifying the smallest fraction. Figure 3b demonstrates that interspecific count variations among algorithms first become detectable at relative abundances below 0.1%, with divergences markedly intensifying below 0.01% thresholds.

In addition, significant differences in beta diversity were observed among samples processed by the four algorithms (Figure 3c, *p* < 0.0001), with DADA2 exhibiting the highest diversity, followed by Deblur, UNOISE3, and UPARSE.

The beta diversity decomposition results (Figure 3d) indicate that different algorithms generate distinct beta diversity components across samples. The UNOISE3 and UPARSE algorithms demonstrate higher similarity in species composition between samples, with values of 0.737 and 0.781, respectively. In these cases, species turnover is lower, and differences in richness are relatively minor, suggesting that the species compositions of samples processed by these two algorithms are more consistent. Conversely, data processed using the DADA2 algorithm show a higher species turnover of 0.643, indicating greater differences in species composition between samples, while similarity and richness differences are lower. The results from the Deblur algorithm fall in between, with a species turnover of 0.442 and a similarity of 0.361, reflecting a more balanced relationship between species turnover and similarity among samples. These findings suggest that different algorithms have distinct impacts on beta diversity among samples, with the DADA2 algorithm tending to introduce greater species turnover, while the UNOISE3 and UPARSE algorithms preserve higher species similarity.

The x-axis represents the transformed values of area, while the y-axis represents the transformed values of species richness. The variable z denotes the slope of the fitted curve, and c is a constant. The R^2^ and *p*-values are used to evaluate the model fit. The gray shaded area indicates the 95% confidence interval, and the naming convention follows the format of algorithm-replication.

### 3.2. The Impact of Model Selection on SARs and the Compatibility Between Algorithms and Models

For the 32 datasets obtained from four algorithms (each algorithm produced eight data points corresponding to the original eight datasets), we utilized 20 SAR models for fitting, resulting in a total of 640 fitting results. The findings indicate that the goodness of fit varies across different models and datasets, with some models exhibiting an R^2^ value of less than 0.6, or even negative. Consequently, models with R^2^ values below 0.6 or negative values were excluded from subsequent analyses as these were considered to represent insignificant relationships.

**Figure 4:**
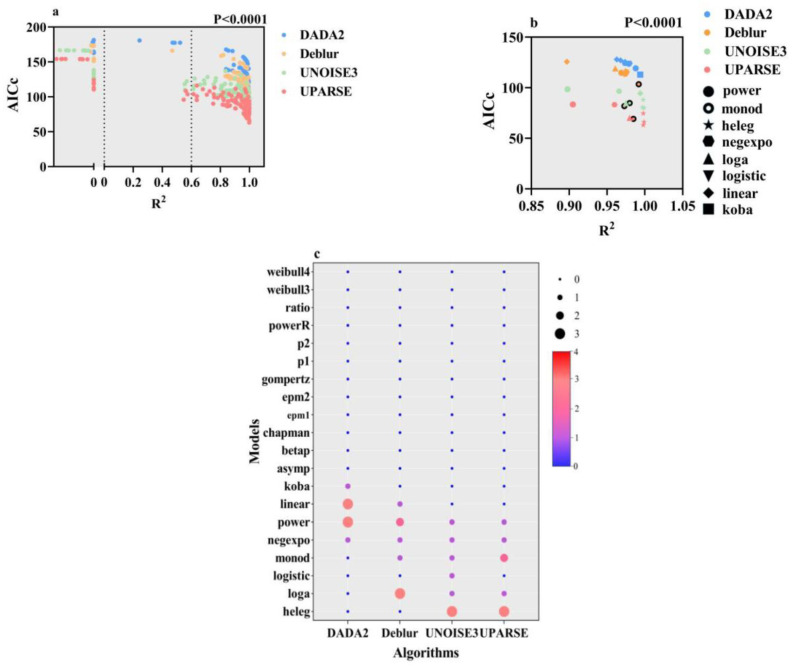
Comparison and verification of algorithm and model fitting results. (**a**) A scatter plot of AICc values and R^2^ for four algorithms fitted with 20 models, where data with R^2^ values less than zero are considered invalid. The *p*-value is calculated using the Kruskal–Wallis method to test for differences. (**b**) A scatter plot of AICc values and R^2^ for the best models across the four algorithms; different colors represent different algorithms, while different shapes denote various models. The *p*-value is also calculated using the Kruskal–Wallis method to test for differences. (**c**) The frequency of the best models under the four algorithms, where larger bubbles indicate a greater number of optimal fitting occurrences for each model.

Of the 640 fitting results, 116 fits with R^2^ values below 0.6 were excluded. Among these, UNOISE3 accounted for 31 fits with R^2^ less than 0.6; DADA2 had 29; Deblur had 26; and UPARSE had 30. Additionally, the chapman, gompertz, and p1 models could not be fitted or exhibited R^2^ values less than zero across all four algorithms. In contrast, the linear, negexpo, betap, and powerR models showed inconsistent performance: while occasionally achieving R^2^ values exceeding 0.9 (approaching 1), they sometimes fell below 0.6 or even reached 0 (Figure 4a).

After excluding models with an R^2^ value of less than 0.6, we further assessed the goodness of fit of the remaining models using corrected Akaike Information Criterion (AICc) values (where a smaller AICc value indicates a better fit). The results indicate that the best-fitting models for datasets generated by different algorithms are significantly different (*p* < 0.0001). Overall, the power model demonstrates the most consistent and superior performance among the four algorithms, particularly under the DADA2 and Deblur algorithms, where lower AICc values indicate improved fits. Conversely, under the UNOISE3 and UPARSE algorithms, the heleg model performs exceptionally well, emerging as the optimal model for these algorithms. Additionally, the performance of models such as linear, negexpo, and Monod varies considerably across different algorithms, with some models fitting well on certain datasets while performing poorly on others.

## 4. Discussion

This study found that employing various high-throughput sequencing data processing algorithms and selecting different fitting models can significantly impact SAR studies of microorganisms. The same sequencing data can yield different species diversity results depending on the algorithms used. Additionally, the compatibility differences between various algorithms and fitting models result in varying fitting outcomes.

First, we conducted a fitting analysis of 32 datasets derived from four different algorithms using a power function model with logarithmic transformation. The results indicated that the SAR slopes fitted under different algorithms exhibited significant differences (*p* < 0.0001). As a core parameter in SAR analysis, the slope reveals the strength of the relationship between species richness and sample area. In this study, the range of slope variation among algorithms was between 0.6209 and 0.8047, suggesting that differences in algorithms significantly impact the characterization of SAR patterns. Particularly when the slope approaches zero—indicating a flat SAR pattern—algorithm discrepancies may obscure weak SAR signals, complicating the identification of specific diversity patterns. This is especially relevant for microbial communities where the weakening or disappearance of SAR patterns is closely linked to algorithm selection. Certain algorithms may be more effective at revealing microbial SAR patterns, while others may diminish or obscure the visibility of such patterns. The study confirmed that the choice of algorithm indeed influences the results of SAR fitting.

To investigate the reasons behind the differences in SAR fitting results produced by various algorithms, this study compared four algorithms based on microbial diversity, species richness, sample composition structure, and beta diversity among samples. The findings revealed significant variations in the number of sequences retained and species richness across the different algorithms. These results were not unexpected, being primarily due to differences in how low-abundance sequences are processed by various algorithms. Specifically, the sequence processing logic of these four algorithms can be categorized into two major groups. The first group includes algorithms that cluster sequences at a 97% similarity threshold to generate Operational Taxonomic Units (OTUs), such as UPARSE [12] The second group consists of algorithms that forgo clustering logic and employ different denoising methods to generate Amplicon Sequence Variants (ASVs), including DADA2, Deblur, and UNOISE3. UPARSE utilizes a lower sequence resolution to reduce noise, resulting in decreased overall richness and fewer low-abundance species [13,14,15,16,18]. The DADA2 algorithm utilizes a probabilistic model to estimate sequencing errors and correct reads, thereby inferring true sequence variants. This precision-oriented approach enables it to identify a greater number of low-frequency variants, making it particularly effective in detecting low-abundance species. In contrast, the Deblur algorithm focuses on preserving high-quality, longer sequences, which maintain high resolution; as a result, it retains the fewest sequences while achieving the second-highest total species richness. The UNOISE3 algorithm employs a different quality control method compared to DADA2 and Deblur, recognizing smaller sequence variations as noise and eliminating them. This leads to a reduction in species richness and low-abundance species. The varying strategies employed by different algorithms lead to inconsistencies in alpha diversity, which subsequently affect the slope of the algorithms. This study demonstrates that the inconsistent treatment of low-abundance species by various algorithms directly influences the estimation of species richness and the fitting results of Species–Area Relationships (SARs). Different algorithms often strive to achieve a balance between denoising and preserving biodiversity to enhance data accuracy and accurately estimate species richness. Notably, the DADA2 algorithm, due to its heightened sensitivity to low-abundance species, exhibits a steeper SAR slope, highlighting its effectiveness in revealing microbial SAR patterns. This further underscores the significance of low-abundance species in SAR analysis.

Different algorithms that employed various balancing strategies resulted in distinct beta diversity outcomes. The findings from the diversity difference study revealed that these algorithms produced varying beta diversities (i.e., differences in species richness) between samples. Overall, the beta diversity patterns observed across the four algorithms were consistent with the patterns of SAR slopes. We hypothesize that the beta diversity differences introduced by the algorithms’ balancing strategies may be a key contributor to the observed differences in SAR slopes. To verify this hypothesis, we conducted a more detailed analysis of the beta diversity difference patterns. Our decomposition analysis confirmed that the UNOISE3 and UPARSE algorithms, due to their lower resolution and higher rates of noise removal, somewhat homogenized the community composition differences between samples, leading to increased similarity among them. In contrast, DADA2 and Deblur, which offer higher accuracy and better retention of low-frequency sequences, resulted in greater species turnover rates between samples, thereby decreasing their similarity. Some studies suggest that SAR encompasses both species turnover and spatial nesting (i.e., beta diversity differences). Consequently, the results of this study indicate that the beta diversity differences arising from the use of different algorithms significantly influence the variations in SAR slopes of the generated datasets.

Secondly, previous studies have suggested that the choice of different models can influence the SAR effect. This study further investigates the compatibility issues between algorithms and models, aiming to identify an optimal combination. Our analysis of eight datasets across four algorithms using twenty models reveals that no single model provides the best fit for all datasets associated with the same algorithm. Furthermore, data generated by each algorithm tend to align more closely with specific models, and the optimal model varies across different datasets. Specifically, DADA2 and Deblur, which generate higher overall species richness and introduce a greater number of species along with increased species turnover rates between samples, are best represented by the classic power model. However, the power model performs better in the DADA2 algorithm, while the loga model performs better in the Deblur algorithm. In contrast, UPARSE and UNOISE3, which exhibit relatively lower resolution and introduce fewer low-abundance species and beta diversity, are better suited to the heleg and monod models. Additionally, different models may excel at addressing various data characteristics, such as the retention of rare species, distribution of species richness, and diversity differences between samples. In summary, each algorithm aims to strike a balance between species retention and removal. Each fitting model has specific application scenarios, constrained by its design intent and the attributes of the target data. Consequently, the compatibility of a dataset generated by a particular algorithm with a specific model does not guarantee that all datasets produced by the same algorithm will be equally suitable for that model. When the objectives of an algorithm align with those of a model, the model fit improves; otherwise, it may result in a lower goodness-of-fit, indicating weaker SAR signals. In this study, the chapman, gompertz, and p1 models were not utilized under any data conditions, likely due to their more specialized focuses, which do not align with the objectives of the algorithms.

Based on this study, we recommend utilizing the DADA2 algorithm, which can identify a greater number of species, along with a power model that is compatible with this algorithm and suitable for high species turnover rates. Furthermore, this study did not consider the effects of varying parameter thresholds of algorithms, different annotation databases, or different sequencing fragments on species richness and, indirectly, on SARs. Therefore, when conducting microbial SAR studies, researchers should flexibly select the most appropriate processing algorithms and fitting models based on specific data characteristics and research objectives to ensure the scientific integrity of the analysis results and the rationality of the interpretations. In future analyses, researchers should examine the synergistic effects of algorithms and models in greater detail to obtain more reliable and profound ecological insights. 

Through literature searches in databases such as Web of Science and PubMed using the keyword “species-area relationship”, we observed limited microbial SAR studies. Existing microbial SAR research has focused on amphibian skin microbiomes [29], human gut microbiomes [30], or alternative frameworks like Species–Time Relationships [31] and Diversity–Area Relationships [32]. However, these datasets cannot exclude confounding effects from environmental heterogeneity and community succession processes on SAR patterns, hindering our systematic evaluation of algorithms and models. Furthermore, while abundant microbial sequencing data exist, most lack explicit habitat area metadata. Although a few studies include habitat area data [33], they remain susceptible to sampling effects and habitat background influences. We anticipate developing a universal methodology for microbial diversity studies, with future work involving multi-scenario data collection and model training.

In conclusion, this study addresses critical challenges in contemporary microbial Species–Area Relationship research by elucidating the dual impacts of algorithms and models. The optimized combination scheme of the DADA2 algorithm and power model proposed here significantly enhances the reliability of microbial diversity–spatial pattern analysis. This advancement holds substantial methodological value for refining microbial biogeography theory and guiding microbial conservation strategy formulation. We emphasize the importance of addressing compatibility between sequence processing algorithms and fitting models in future studies, advocating for expanded fitting attempts to derive ecologically robust conclusions.

## Data Availability

The data presented in this study are openly available in GSA at https://ngdc.cncb.ac.cn/gsa/browse/CRA008829 (accessed on 23 May 2023), reference number CRA008829.
